# Higher Risk of Loss to Follow-up Among Transgender People With HIV in Italy: Findings From the ICONA Cohort

**DOI:** 10.1093/ofid/ofag640

**Published:** 2026-10-08

**Authors:** Davide Moschese, Marco Muccio, Giuseppe Lapadula, Valentina Mazzotta, Elena Bruzzesi, Andrea Poloni, Massimo Cernuschi, Alessandra Rodano', Silvia Nozza, Antonio Di Biagio, Sergio Lo Caputo, Cristina Mussini, Alessandro Cozzi-Lepri, Roberto Rossotti, Andrea Giacomelli, ICONA Cohort Study Group, A d'Arminio Monforte, A Antinori, S Antinori, A Castagna, M Cernuschi, L Cosmaro, A Di Biagio, E Dibrino, E Girardi, S Lo Caputo, G C Marchetti, C Mussini, E Quiros-Roldan, L Sarmati, B Suligoi, C Torti, A d’Arminio Monforte, A Antinori, A Castagna, F Ceccherini-Silberstein, A Cingolani, A Cozzi-Lepri, A Di Biagio, R Gagliardini, A Giacomelli, E Girardi, A Gori, S Lo Caputo, G Marchetti, F Maggiolo, C Mussini, M Puoti, C F Perno, A Tavelli, A Antinori, A Bandera, S Bonora, A Calcagno, A Castagna, F Ceccherini-Silberstein, A Cervo, A Cingolani, P Cinque, A Cozzi-Lepri, A d’Arminio Monforte, A De Vito, A Di Biagio, A Fortunato, R Gagliardini, A M Geretti, A Giacomelli, E Girardi, N Gianotti, A Gori, G Guaraldi, S Lanini, G Lapadula, M Lichtner, A Lai, S Lo Caputo, G Madeddu, F Maggiolo, V Malagnino, I Mastrorosa, G Marchetti, A Mondi, V Mazzotta, C Muccini, C Mussini, S Nozza, C F Perno, S Piconi, C Pinnetti, M C Poliseno, M Puoti, E Quiros Roldan, R Rossotti, S Rusconi, M M Santoro, A Saracino, L Sarmati, V Spagnuolo, N Squillace, V Svicher, L Taramasso, C Tincati, C Torti, A Vergori, A Cozzi-Lepri, S De Benedittis, I Fanti, M Giotta, S Lanini, N Lentini, M Muccio, R Pastorino, A Rodano’, A Roen, A Tavelli, S Bazzichetto, M Cernuschi, L Cosmaro, A Perziano, V Calvino, D Russo, M Farinella, N Policek, V L Del Negro, S Carrara, S Graziano, G Prota, H Sadeghi, V Sala, S Truffa, D Vincenti, R Rovito, A Giacometti, A Costantini, V Barocci, A Saracino, C Santoro, E Milano, L Comi, C Suardi, P Viale, L Badia, S Cretella, E M Erne, A Pieri, E Quiros Roldan, E Focà, S Minniti, B Menzaghi, C Abeli, L Chessa, F Pes, P Maggi, L Alessio, G Nunnari, B M Celesia, J Vecchiet, K Falasca, A Pan, S Dal Zoppo, D Segala, A Bartoloni, B Borchi, C Costa, A Castellaccio, S Lo Caputo, A Narducci, M Bassetti, E Pontali, S Blanchi, N Bobbio, C Del Borgo, R Marocco, G Mancarella, S Piconi, C Molteni, S Rusconi, G Canavesi, P Colletti, G Pellicanò, Y Russotto, G Marchetti, S Antinori, A Gori, M Puoti, A Castagna, A Bandera, V Bono, G Bozzi, M V Cossu, V Iannone, R Lolatto, M C Moioli, G Carrozzo, N Capra, M Augello, D Moschese, C Mussini, M Menozzi, P Bonfanti, G Lapadula, V Sangiovanni, I Gentile, V Esposito, N Coppola, F M Fusco, G Di Filippo, V Rizzo, N Sangiovanni, S Martini, A M Cattelan, D Leoni, A Cascio, C Iaria, M Trizzino, R Gulminetti, D Francisci, E Schiaroli, G Parruti, F Sozio, D Messeri, S I Bonelli, C Lazzaretti, R Corsini, A Antinori, C Torti, G D’Ettorre, L Sarmati, A Latini, A Cingolani, G Micheli, S Lamonica, M Capozzi, M Camici, I Mezzaroma, M Rivano Capparuccia, G Iaiani, C Stingone, L Gianserra, J Paulicelli, M M Plazzi, L Bortolani, M Fusto, I Coledan, G Madeddu, G Moi, M Fabbiani, F Montagnani, A Franco, R Fontana Del Vecchio, D Francisci, C Di Giuli, G C Orofino, G Calleri, G Di Perri, S Bonora, D Meloni, C Tascini, A Londero, G Battagin, S Nicolè, G Starnini, S Dell’Isola

**Affiliations:** Department of Biomedical and Clinical Sciences, Università degli Studi di Milano, Milan, Italy; Department of Infectious Diseases, ASST Fatebenefratelli Sacco, Milan, Italy; ICONA Foundation, Milan, Italy; IRCCS San Gerardo dei Tintori, University of Milano-Bicocca, Department of Infectious Diseases, Monza, Italy; Clinical Infectious Diseases Department, National Institute for Infectious Diseases Lazzaro Spallanzani IRCCS, Rome, Italy; Department of Infectious Diseases, IRCCS San Raffaele Scientific Institute, Vita-Salute San Raffaele University, Milan, Italy; Department of Infectious Diseases, ASST Fatebenefratelli Sacco, Milan, Italy; A.S.A. Onlus-Association of Solidarity AIDS, Milan, Italy; ICONA Foundation, Milan, Italy; Department of Infectious Diseases, IRCCS San Raffaele Scientific Institute, Vita-Salute San Raffaele University, Milan, Italy; Department of Health Science (DISSAL), Infectious Disease Clinic, IRCCS Ospedale Policlinico San Martino, University of Genova, Genoa, Italy; Department of Clinical and Surgical Sciences, Clinic of Infectious Diseases, University of Foggia, Foggia, Italy; Azienda Ospedaliero-Universitaria di Modena, University of Modena, Reggio Emilia, Italy; Department of Infectious Diseases, Policlinico di Modena, Modena, Italy; Centre for Clinical Research, Epidemiology, Modelling and Evaluation (CREME), Institute for Global Health, UCL, London, UK; Department of Infectious Diseases, ASST Grande Ospedale Metropolitano Niguarda, Milan, Italy; Department of Biomedical and Clinical Sciences, Università degli Studi di Milano, Milan, Italy; Department of Infectious Diseases, ASST Fatebenefratelli Sacco, Milan, Italy

**Keywords:** cisgender, clinical outcomes, gender identity, loss to follow-up, transgender

## Abstract

**Background:**

Official data on transgender people with human immunodeficiency virus (PWH) in Italy are lacking because gender identity is not recorded in national reports. We aimed to characterize transgender PWH in Italy and assess retention in care and clinical outcomes.

**Methods:**

We included PWH enrolled between 2010 and 2023 in 40 Italian Cohort Naive Antiretrovirals centers where gender identity could be reliably ascertained. Transgender PWH were compared with cisgender participants. Univariable and multivariable regression models assessed the association between gender identity and (1) loss to follow-up (LTFU; no visit or exam for ≥18 months) using Fine–Gray models and (2) a composite clinical endpoint including mortality, major adverse cardiovascular events, cancer, acquired immunodeficiency syndrome–defining events, and end-stage kidney and liver disease using Cox models. Sensitivity analyses were restricted to individuals who initiated antiretroviral therapy (ART) from 2016 onward.

**Results:**

Overall, 256 transgender and 9922 cisgender PWH were included. Transgender PWH were younger (median 32 vs 39 years, *P* < .0001), more often assigned male at birth (99.6% vs 82.4%, *P* < .0001). Transgender PWH had a higher unadjusted risk of LTFU (subdistribution hazard ratio [sHR] 1.88, 95% confidence interval [CI] 1.60–2.22), confirmed after adjustment (adjusted sHR [asHR] 1.41, 95% CI 1.18–1.68) and in the post-2016 subgroup (asHR 1.39, 95% CI 1.11–1.77). Composite endpoint analyses were inconclusive (transgender PWH vs cisgender PWH: overall adjusted hazard ratio [aHR] 1.30 [95% CI .79–2.14], *P* = .293; post-2016 aHR 1.69 [95% CI .91–3.16], *P* = .098).

**Conclusions:**

Transgender PWH had a substantially higher risk of LTFU than cisgender participants in this national Italian cohort. Tailored interventions are needed to improve retention and health outcomes.

## Teaser

Transgender people with HIV had a substantially higher risk of loss to follow-up than cisgender people despite universal care in Italy, underscoring the need for tailored strategies to improve retention.

Ensuring timely access to antiretroviral therapy and sustaining long-term engagement in treatment among people with human immunodeficiency virus (PWH) remains a cornerstone of global HIV control efforts and reduces the risk of HIV-related morbidity and mortality [1]. The HIV care continuum has become a key framework for monitoring progress toward epidemic control and for achieving the Joint United Nations Programme on HIV/AIDS 95–95–95 targets for 2025 [2]. Despite substantial progress in most European countries, inequalities persist among key populations, including transgender people [3].

Transgender people, and in particular transgender women, defined as individuals assigned male at birth who identify as women, are disproportionately affected by HIV worldwide [4]. While Europe has made significant advances in HIV prevention and treatment, the epidemiological picture for transgender people remains fragmented limiting the ability to identify gaps in testing, treatment uptake, and retention in care [5, 6].

The available evidence suggests that transgender women in Europe experience suboptimal HIV care outcomes compared with cisgender PWH [2, 6]. Observational studies indicate lower rates of retention in care, ART initiation, and viral suppression among transgender PWH, often driven by intersecting structural and social factors [7]. Discrimination within healthcare settings, lack of culturally competent or gender-affirming services, and limited access to hormone therapy in HIV clinics contribute to disparities in engagement across the HIV care continuum [8].

Loss to follow-up (LTFU) remains a major barrier to achieving sustained viral suppression and accurately assessing progress toward HIV care targets. Longitudinal data that link gender identity with clinical outcomes are scarce, and official data on transgender PWH in Italy are lacking, as gender identity is not recorded in national reports. Thus, we aimed to characterize transgender PWH in the Italian Cohort Naive Antiretrovirals (ICONA) cohort, which included more than one-third of newly diagnosed PWH in Italy [9], and assess their retention in care and clinical outcomes.

## METHODS

### Study Design

ICONA is a nationwide multicenter observational prospective cohort, established in 1997, including PWH aged >18 years who are ART-naive at the time of enrollment in 91 Italian infectious diseases centers. Further details about the cohort are available elsewhere [10].

For the present study, gender identity was ascertained using a combination of information recorded in the dedicated ICONA electronic case-report form (eCRF), when available, and review of clinical records in centers where gender identity could be reliably reconstructed. The ICONA eCRF records sex assigned at birth and includes the following gender identity categories: cisgender, transgender, nonbinary, and other. Of the 91 ICONA centers, 40 had sufficiently systematic or reliably reconstructable gender identity information and were therefore included in the present analysis; participants from centers where this information could not be reliably ascertained were excluded. We included PWH enrolled between 1 January 2010 and 31 December 2023. Participants were followed until death, administrative censoring due to discontinuation of ICONA cohort participation by individual choice, or 31 December 2024, whichever occurred first.

### Patient Consent Statement

The ICONA Foundation study was approved by the local Ethics Committees of each center. The latest amendment was centrally approved by the Lazio Area 4 Territorial Ethics Committee on 26 January 2026 (approval no. 07-2026). All participants provided written informed consent for study participation and processing of personal data, in accordance with the ethical principles of human experimentation as outlined by the Declaration of Helsinki (last amended in October 2013) and applicable legislation.

Our current study proposal was shared with community representatives, which are part of ICONA Foundation advisory board. The reporting of the study adheres to the Strengthening of the Reporting of Observational Studies in Epidemiology guidelines [11].

### Follow-up, Variables, Definitions, and Outcomes

Baseline was defined as the date of enrollment in the ICONA cohort, and participants were followed thereafter.

Data of the ICONA cohort were collected in an electronic eCRF (https://www.icona.org/). The variables considered for the present analysis were calendar period of cohort enrollment—categorized into 2010–2015 and 2016–2023 (with 2016 chosen as the cutoff year due to the implementation of universal ART recommendations in Italy), age (in years), sex assigned at birth, gender identity (transgender man and woman or cisgender man and woman), place of birth (Italian-born vs foreign-born), risk factor for HIV acquisition (people who inject drugs [PWID] vs all other exposure categories), CD4+ T-cell count at enrollment (<200, 200–349, 350–499, and ≥500 cells/μL), Hepatitis C virus (HCV) antibody status (positive and negative), HBsAg positivity (positive and negative), smoking habits (smoker, nonsmoker and unknown) and comorbidities (acquired immunodeficiency syndrome [AIDS]–defining events, major cardiovascular events (MACEs) (myocardial infarction, stroke, or coronary revascularization), cancer (non–AIDS-defining malignancies), diabetes, end-stage kidney and liver disease, hypertension, and death [12].

Comorbidities recorded within 15 days after baseline were considered prevalent and classified as baseline comorbidities. This 15-day window was defined a priori and is routinely used in ICONA analyses to account for possible misclassification of the exact recording date of preexisting comorbidities in an observational database.

We considered the following outcomes:

1. Loss to follow-up (LTFU): absence of any clinical or laboratory data recorded in the ICONA cohort for a period of at least 18 months, in the absence of a documented reason for follow-up discontinuation.
2. Definitive LTFU: LTFU with no subsequent resumption of recorded follow-up through the end of the observation period.
3. Composite clinical endpoint: first occurrence of death, MACE, cancer, AIDS-defining events, and end-stage liver and kidney disease [12].

### Statistical Analysis

Demographic and clinical characteristics at enrollment were summarized as median (interquartile range [IQR]) for continuous variables and as count (percentage) for categorical variables. Comparisons between transgender and cisgender individuals were performed using the Chi-square test or Fisher's exact test for categorical variables and the Wilcoxon rank-sum test for continuous variables. The distribution of baseline CD4+ T-cell counts was also graphically explored using histograms stratified by gender identity. In addition, we performed a descriptive comparison restricted to transgender women and cisgender women.

The association between gender identity and LTFU was assessed using Fine–Gray competing-risk regression, with death treated as a competing event. Univariable models were first fitted, followed by multivariable models including gender identity and relevant baseline covariates. The primary adjusted model additionally included study center as a fixed effect to account for between-center heterogeneity [13]. The multivariable analysis was also repeated after restricting the comparison to transgender women and cisgender women and, separately, by including an interaction term between gender identity and place of birth (Italian-born vs foreign-born) to explore potential effect modification by migration background. The association between gender identity and the composite clinical endpoint was explored using Cox proportional hazards regression. Univariable and multivariable models were fitted including relevant baseline covariates. Participants with a history of one component of the composite endpoint before baseline were not considered at risk for recurrence of that same component but remained at risk for all other components of the composite endpoint. To account for the multicentre structure of the data, the adjusted model was additionally fitted using a shared frailty term for study center [13]. Further exploratory models were fitted excluding comorbidity-related covariates, namely previous diabetes and hypertension (model A), and excluding participants with a previous component event together with the corresponding baseline covariates (model B). The multivariable analyses were also repeated after restricting the comparison to transgender women and cisgender women and, separately, by including an interaction term between gender identity and place of birth. Cumulative incidence functions stratified by gender identity were generated for LTFU, and differences between groups were assessed using Gray's test. For the composite clinical endpoint, Kaplan–Meier curves were generated and compared using the log-rank test. Definitive and nondefinitive LTFU were summarized descriptively among participants who experienced LTFU and compared between gender identity groups using the chi-square test in an exploratory analysis. Missing baseline CD4+ T-cell counts were handled using multiple imputation by chained equations with predictive mean matching. Baseline CD4 count was the only continuous covariate included in the multivariable models that required imputation; age had no missing values. The imputation model included age, sex assigned at birth, country of birth (Italy vs other), year of ART initiation, previous AIDS diagnosis, and injecting drug use as predictors. Five imputed datasets were generated, and estimates were combined according to Rubin's rules [14]. The imputation procedure was performed separately for the overall study population and for the sensitivity analysis restricted to individuals who initiated ART from 2016 onward. Outcome variables were not included in the imputation model. Imputed CD4 values were used in the multivariable analyses of the composite clinical endpoint. A *P* value of ≤.05 was considered statistically significant, and all tests were 2-sided. Analyses were conducted with SAS software version 9.4 (SAS Institute Inc., Cary, NC, United States).

### Sensitivity Analyses

As a sensitivity analysis, we restricted the study population to individuals who initiated ART from 2016 onward. In this subgroup, demographic and clinical characteristics at ART initiation were summarized and compared by gender identity, and all primary and exploratory analyses were repeated using the same analytical approach as in the overall study population.

To assess potential selection related to the availability of gender identity information across centers, we also performed an exploratory comparison of the main demographic and clinical characteristics of participants from included vs excluded centers among individuals who initiated ART from 2016 onward. This restriction was chosen because, from 2016 onward, ART was recommended irrespective of CD4+ T-cell count, allowing a more comparable contemporary cohort [15].

## RESULTS

A total of 10 178 PWH were included, of whom 256 were transgender and 9922 were cisgender. Among transgender participants, 255 were transgender women and 1 was a transgender man; no participants were classified as nonbinary or other. Characteristics of the study population are reported in Table 1. Compared with cisgender PWH, transgender PWH were younger (median age 32 [IQR 28–41] vs 39 [IQR 31–48] years, *P* < .0001), were less frequently born in Italy (8.2% vs 74.9%, *P* < .0001), and had a lower prevalence of PWID (2.3% vs 5.7%, *P* = .022). Smoking was more frequent among transgender participants (53.1% vs 46.2%), although this difference was not statistically significant (*P* = .087), whereas HBsAg positivity was more frequent among transgender than cisgender participants (5.9% vs 2.8%, *P* = .014). Transgender PWH had a higher median CD4 cell count at enrollment than cisgender PWH (411 [IQR 237–581] vs 348 cell/mm^3^ [IQR 151–538], *P* < .0001).

**Table 1.** Demographic and Clinical Characteristics at Enrollment According to Gender Identity

Variables	Total n = 10 178	Transgender n = 256	Cisgender n = 9922	*P* Value
Age (y), median (IQR)	39 (31–48)	32 (28–41)	39 (31–49)	<.0001
Male sex assigned at birth, n (%)	8429 (82.8)	255 (99.6)	8174 (82.4)	<.0001
Italian origin, n (%)	7449 (73.2)	21 (8.2)	7428 (74.9)	<.0001
PWID, n (%)	570 (5.6)	6 (2.3)	564 (5.7)	.022
Smoking habit, n (%)				.087
Yes	4721 (46.4)	136 (53.1)	4585 (46.2)	
No	4426 (43.5)	96 (37.5)	4330 (43.6)	
Unknown	1031 (10.1)	24 (9.4)	1007 (10.2)	
Comorbidities, n (%)				
Previous AIDS diagnosis	1160 (11.4)	24 (9.4)	1136 (11.5)	.302
Previous MACE event	60 (0.6)	0 (0.0)	60 (0.6)	.407
Previous cancer diagnosis	149 (1.5)	1 (0.4)	148 (1.5)	.189
Previous kidney/liver event	11 (0.1)	0 (0.0)	11 (0.1)	1.000
Previous diabetes	192 (1.9)	3 (1.2)	189 (1.9)	.637
Previous hypertension	514 (5.1)	6 (2.3)	508 (5.1)	.045
HCVAb positivity				.255
Yes	500 (4.9)	9 (3.5)	491 (5.0)	
No	9244 (90.8)	240 (93.8)	9004 (90.8)	
Unknown	434 (4.3)	7 (2.7)	427 (4.3)	
HbsAg positivity				.014
Yes	293 (2.9)	15 (5.9)	278 (2.8)	
No	9512 (93.5)	233 (91.0)	9279 (93.5)	
Unknown	373 (3.7)	8 (3.1)	365 (3.7)	
Year of enrollment, n (%)				<.001
2010–2015	4186 (41.1)	78 (30.5)	4108 (41.4)	
2016–2023	5992 (58.9)	178 (69.5)	5814 (58.6)	
CD4, cells/mm^3^, median (IQR)	n = 9458 350 (155–540)	n = 246 411 (237–581)	n = 9212 348 (151–538)	<.0001
CD4, cells/mm^3^, n (%)				<.001
<200	2854/9458 (30.2)	53/246 (21.5)	2801/9212 (30.4)	
200–349	1863/9458 (19.7)	38/246 (15.5)	1825/9212 (19.8)	
350–499	1965/9458 (20.8)	65/246 (26.4)	1900/9212 (20.6)	
≥500	2776/9458 (29.4)	90/246 (36.6)	2686/9212 (29.2)	

The reported *P* values are from the Wilcoxon rank-sum test for continuous variables and Chi-square test or Fisher's exact test for categorical variables. Missing values were treated as an “Unknown” category for categorical variables and reported as counts for continuous variables.

Abbreviations: IQR, interquartile range; n, number; MACEs; major cardiovascular events; PWID, people who inject drugs; HCVAb, hepatitis C virus antibodies.

Among participants with available baseline CD4 counts, 21.5% of transgender and 30.4% of cisgender participants had CD4 counts <200 cells/µL. Previous AIDS diagnoses were present in 9.4% and 11.5%, respectively. The full distribution of baseline CD4 counts by gender identity is shown in Supplementary Figure 1. Similar descriptive patterns were observed among individuals who initiated ART from 2016 onward (Supplementary Table 1) and in the comparison restricted to transgender women and cisgender women (Supplementary Table 2).

In the exploratory comparison of participants from included vs excluded centers among individuals who initiated ART from 2016 onward, the 2 groups were broadly similar with respect to age, country of birth, comorbidity burden, and baseline CD4 count, although somewhat greater missingness for selected variables was observed among participants from excluded centers (Supplementary Table 3).

### Loss to Follow-up

Overall, 4026 participants experienced LTFU during follow-up. Among transgender participants, 136 experienced LTFU, of whom 96 were classified as definitive LTFU and 40 as nondefinitive LTFU. Among cisgender participants, 3890 experienced LTFU, including 2665 definitive and 1225 nondefinitive LTFU. Among individuals who initiated ART from 2016 onward, 73 transgender participants experienced LTFU (51 definitive and 22 nondefinitive), compared with 1691 cisgender participants (1252 definitive and 439 nondefinitive). Among participants experiencing LTFU, the distribution of definitive vs nondefinitive LTFU did not differ significantly by gender identity either in the overall population (*P* = .697) or among individuals who initiated ART from 2016 onward (*P* = .426) (Supplementary Table 4).

The 4-year cumulative incidence of LTFU was higher among transgender than cisgender participants, both in the overall population (55% [95% confidence interval (CI) 47%–61%] vs30% [95% CI 29%–31%]) and among individuals who initiated ART from 2016 onward (52% [95% CI 42%–61%] vs33% [95% CI 31%–34%]). Differences between the cumulative incidence functions were statistically significant in both analyses (Gray's test *P* < .001) (Figure 1*A* and 1*C*).

**Figure 1.**
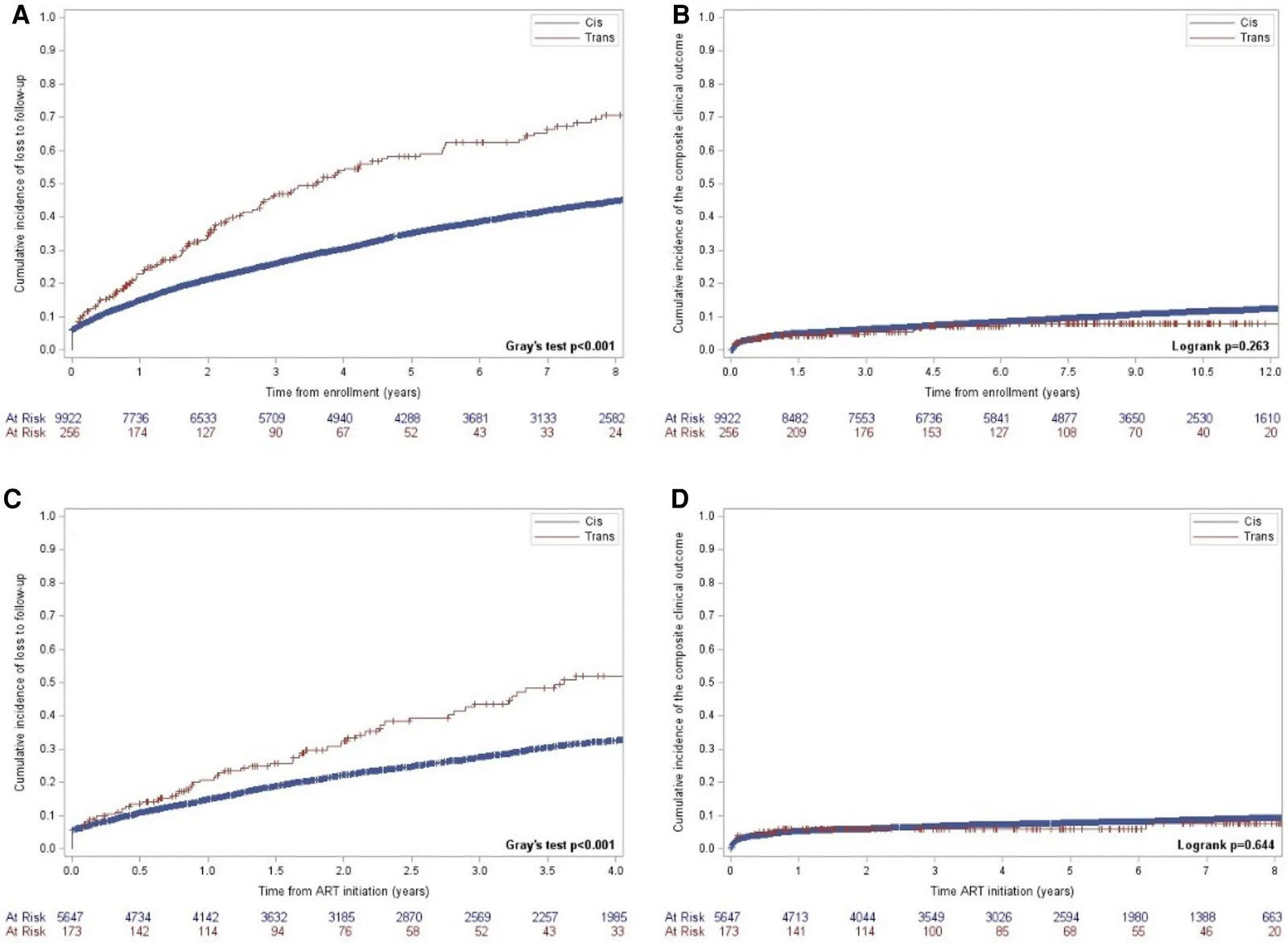
Cumulative incidence of loss to follow-up (*A* and *C*) and composite clinical outcome (*B* and *D*), according to gender identity, for the overall population (*A* and *B*) and restricted to people with HIV (PWH) who initiated ART from 2016 (*C* and *D*) populations.

In the overall population, transgender participants had a higher unadjusted subdistribution hazard of LTFU than cisgender participants (subdistribution hazard ratio [sHR] 1.88, 95% CI 1.60–2.22; *P* < .0001). In the primary adjusted model, which also accounted for study center, transgender gender identity remained associated with a higher risk of LTFU (adjusted sHR [asHR] 1.41, 95% CI 1.18–1.68; *P* = .0001). The association remained evident among individuals who initiated ART from 2016 onward (asHR 1.39, 95% CI 1.11–1.77; *P* = .005) (Table 2).

**Table 2.** Univariable and Multivariable Models to Assess the Effect of Gender Identity on Risk of Loss to Follow-up

	Univariable		Multivariable		Multivariable Including Center^a^	
Overall^b^	sHR (95% CI)	*P* Value	sHR^c^ (95% CI)	*P* Value	sHR (95% CI)	*P* Value
Transgender (vs cisgender)	1.88 (1.60–2.22)	<.0001	1.32 (1.12–1.57)	.001	1.41 (1.18–1.68)	.0001

Restricted to 2016 onward^d^	sHR (95% CI)	*P* Value	sHR^e^ (95% CI)	*P* Value	sHR (95% CI)	*P* Value
Transgender (vs cisgender)	1.71 (1.37–2.13)	<.0001	1.26 (1.01–1.58)	.044	1.39 (1.11–1.77)	.005

The reported *P* values are from the Fine–Gray regression analysis with death as competing event.

Abbreviations: CI, confidence interval; sHR, subdistribution hazard ratio.

^a^See “Statistical Analysis” section.

^b^4026 events.

^c^Adjusted for years of enrollment (2010–2015 vs 2016–2023), age, previous AIDS diagnosis, country of birth, and people who inject drugs.

^d^1764 events.

^e^Adjusted for age, previous AIDS diagnosis, country of birth, and people who inject drugs.

In the additional analysis restricted to transgender women and cisgender women, the adjusted association remained in the same direction and was statistically significant in the overall population (sHR 1.27, 95% CI 1.06–1.53; *P* = .012). Among individuals who initiated ART from 2016 onward, the point estimate was similar in direction but more imprecise and did not reach statistical significance (sHR 1.24, 95% CI .96–1.59; *P* = .098) (Supplementary Table 5).

Country of birth was included as an adjustment covariate in the multivariable models. There was no statistical evidence of effect modification of the association between gender identity and LTFU by place of birth, either in the overall population (interaction *P* = .908) or among individuals who initiated ART from 2016 onward (interaction *P* = .649).

### Composite Clinical Outcome

Overall, 961 composite clinical events occurred, including 17 events among transgender participants. Among individuals who initiated ART from 2016 onward, 433 events occurred, including 11 among transgender participants. The distribution of individual components of the composite endpoint is reported in Supplementary Tables 6 and 7.

The 4-year probability of experiencing the composite clinical endpoint did not differ significantly between transgender and cisgender participants in either the overall population (5% [95% CI 3%–9%] vs 7% [95% CI 6%–8%], log-rank *P* = .263) or among individuals who initiated ART from 2016 onward (6% [95% CI 3%–11%] vs 7% [95% CI 7%–8%], log-rank *P* = .644) (Figure 1*B* and 1*D*).

In the univariable Cox model, transgender participants had a lower, nonsignificant crude hazard of the composite endpoint than cisgender participants (hazard ratio [HR] 0.76, 95% CI .47–1.23; *P* = .265). After adjustment for demographic and clinical characteristics and accounting for center-level heterogeneity using a shared frailty model, the point estimate crossed the null and shifted toward a higher hazard among transgender participants (adjusted HR [aHR] 1.30, 95% CI .79–2.14; *P* = .293). A similar pattern was observed among individuals who initiated ART from 2016 onward (crude HR 0.87, 95% CI .48–1.58; *P* = .645; adjusted shared frailty aHR 1.69, 95% CI .91–3.16; *P* = .098) (Table 3).

**Table 3.** Univariable and Multivariable Models to Assess the Effect of Gender Identity on the Risk of a Composite Clinical Outcome Composed by Mortality, MACEs, Cancer Incidence, AIDS Progression, and Other Severe Non-AIDS Events

	Univariable	Multivariable			Shared Frailty^a^	Model A^a^		Model B^a^		
Overall^b^	HR (95% CI)	*P* Value	aHR^c^ (95% CI)	*P* Value	aHR (95% CI)	*P* Value	aHR (95% CI)	*P* Value	aHR^d^ (95% CI)	*P* Value
Transgender (vs cisgender)	0.76 (.47–1.23)	.265	1.19 (.73–1.95)	.484	1.30 (.79–2.14)	.293	1.19 (.73–1.96)	.480	1.07 (.62–1.84)	.818

Restricted to 2016 onward^e^	HR (95% CI)	*P* Value	aHR^f^ (95% CI)	*P* Value	aHR (95% CI)	*P* Value	aHR (95% CI)	*P* Value	aHR^g^ (95% CI)	*P* Value
Transgender (vs cisgender)	0.87 (.48–1.58)	.645	1.54 (.83–2.87)	.170	1.69 (.91–3.16)	.098	1.54 (.83–2.86)	.171	1.58 (.82–3.05)	.171

The reported *P* values are from the Cox regression analysis.

Abbreviations: aHR, adjusted hazard ratio; CI: confidence interval; HR: hazard ratio.

^a^See “Statistical Analysis” section.

^b^961 events;.

^c^Adjusted for years of enrollment (2010–2015 vs 2016–2023), age, previous AIDS diagnosis, previous MACE, previous cancer, previous kidney/liver event, previous diabetes and hypertension, CD4 count at enrollment, country of birth, HCV and HBsAg positivity, smoking habits, and people who inject drugs.

^d^8827 individuals, 725 events.

^e^433 events.

^f^Adjusted for age, previous AIDS diagnosis, previous MACE, previous cancer, previous kidney/liver event, previous diabetes and hypertension, CD4 count at enrollment, country of birth, HCV and HBsAg positivity, smoking habits, and people who inject drugs.

^g^5030 individuals, 327 events.

Estimates were similar in direction across alternative multivariable models after excluding diabetes and hypertension (model A) and after excluding participants with previous component events together with the corresponding baseline covariates (model B) (Table 3).

In the analysis restricted to transgender women and cisgender women, adjusted estimates were also above 1 but remained imprecise and nonsignificant both overall (aHR 1.26, 95% CI .74–2.16; *P* = .396) and among individuals who initiated ART from 2016 onward (aHR 1.49, 95% CI .75–2.97; *P* = .259) (Supplementary Table 8).

There was no statistical evidence of effect modification by place of birth for the composite clinical endpoint in either the overall population (interaction *P* = .119) or among individuals who initiated ART from 2016 onward (interaction *P* = .791).

## DISCUSSION

In this large multicentre national cohort of PWH in Italy, transgender participants had a substantially higher risk of LTFU than cisgender participants. This association remained after multivariable adjustment including study center and was also observed among individuals who initiated ART from 2016 onward. Nearly all transgender participants were transgender women, and the additional analysis restricted to transgender women and cisgender women showed a similar direction of association, supporting the robustness of the main finding.

The 4-year probability of LTFU was approximately 1.7-fold higher among transgender PWH compared with cisgender PWH in the overall population (55% vs 30%) and remained elevated also after restricting the analysis to the post-2016 period, corresponding to the implementation of universal ART (1.6-fold higher; 52% vs 33%). In the Italian setting, where HIV care is universally accessible and free of charge, these findings indicate that disparities in engagement across the HIV care continuum among transgender individuals persist despite the removal of financial barriers to healthcare access. This suggests that access to care alone is insufficient to ensure sustained engagement and that structural, social, and healthcare-related barriers may contribute to difficulties in sustained retention in care. Notably, these patterns were observed among individuals already linked to care, pointing to challenges in retention, in addition to initial linkage to care. Consistently, similar evidence has been described in other European and North American cohorts [16–18].

Among participants who experienced LTFU, the proportion classified as definitive rather than nondefinitive did not differ significantly by gender identity. This finding suggests that the excess burden of LTFU among transgender participants was not clearly attributable to a greater proportion of permanently unrecorded follow-up once LTFU had occurred. However, these data do not allow us to characterize the frequency or recurrence of individual interruptions in care and therefore should not be interpreted as evidence of recurrent intermittent disengagement. Previous longitudinal cohorts have nevertheless shown that engagement in HIV care among transgender women may vary over time and be influenced by multiple social and healthcare-related factors [17, 19] such as the presence of time-varying and context-dependent barriers to care, like stigma, competing priorities, unstable living conditions or challenges in accessing gender-affirming services within HIV care settings. Such factors may lead to repeated disruptions in engagement, even among individuals who have already been successfully linked to care. From a clinical and public health perspective, this highlights how continuity of care, rather than initial linkage or re-engagement alone, is a key unmet need in this population. Importantly, although ICONA records documented reasons for follow-up discontinuation when known, some participants classified as LTFU may have continued HIV care elsewhere without this information being available to the original center. LTFU should therefore not be considered synonymous with confirmed disengagement from HIV care.

The marked imbalance in country of birth between groups is an important aspect of the interpretation of our findings. Fewer than 10% of transgender participants were Italian-born, compared with 74.9% of cisgender participants, highlighting the substantial overlap between transgender identity and migration background in this cohort. Country of birth was included in the multivariable models to address measured confounding, and no statistical evidence of effect modification by place of birth was observed for LTFU. However, these interaction analyses were limited by the small number of Italian-born transgender participants and should not be interpreted as demonstrating homogeneity of the association across migration groups. Moreover, country of birth is only a crude proxy for migration-related vulnerability and does not capture factors such as duration of residence, legal status, mobility, housing instability, or other social determinants that may influence continuity of care. Migration-related vulnerabilities have previously been associated with poorer HIV care outcomes and LTFU in the Italian setting [20]. Our findings therefore support an intersectional interpretation in which gender identity and migration-related factors may jointly contribute to vulnerability to LTFU.

The analyses of the composite clinical endpoint should be interpreted as exploratory. In crude analyses, transgender participants appeared to have a lower risk of the composite outcome; however, after adjustment, the point estimates crossed the null and shifted toward a higher risk. This change in direction is compatible with confounding by baseline characteristics that differed between transgender and cisgender participants, including younger age and higher baseline CD4 counts among transgender participants, and suggests that the lower crude estimate should not be interpreted as evidence of a protective association. At the same time, adjusted estimates remained imprecise, with wide confidence intervals crossing 1, and did not reach statistical significance. The small number of clinical events among transgender participants further limits inference. Accordingly, these findings should be considered hypothesis-generating rather than evidence of an increased risk of adverse clinical outcomes. Previous prospective evidence has nevertheless documented substantial morbidity and mortality among transgender women with HIV and highlighted the contribution of structural determinants to long-term health outcomes [21].

Several limitations should be considered. First, gender identity was ascertainable in only 40 of the 91 ICONA centers, which may have introduced selection bias and limits the generalizability of our findings. Although participants from included and excluded centers who initiated ART from 2016 onward were broadly similar across several measured characteristics, unmeasured differences between centers cannot be excluded. Second, gender identity was ascertained using both dedicated eCRF information and review of clinical records, which may have introduced some heterogeneity in classification. The way gender identity is measured can materially affect the identification and characterization of transgender populations in observational datasets [22]. Third, despite the availability of documented reasons for follow-up discontinuation in ICONA, some participants classified as LTFU may have continued HIV care elsewhere without this being known to the original center, leading to possible residual misclassification. Fourth, the marked overlap between transgender identity and foreign birth raises the possibility of residual confounding by migration-related and socioeconomic factors not captured in the cohort, including duration of residence in Italy, legal status, mobility, housing instability, and experiences of stigma. Country of birth represents only a crude proxy for these complex migration-related vulnerabilities [20]. In addition, interaction analyses should be interpreted cautiously due to the low number of Italian-born transgender participants. Fifth, information on gender-affirming hormone therapy and access to trans-competent or gender-affirming services was not available, precluding direct assessment of their potential impact on retention in care and clinical outcomes. Consequently, although barriers to gender-affirming care and broader sociocultural factors may contribute to difficulties in sustained engagement in HIV care, their role could not be directly evaluated in the present study. Finally, the relatively small number of transgender participants, and particularly the limited number of clinical events, reduced statistical power for the clinical and subgroup analyses. Dedicated analyses of HIV-specific outcomes, including viral suppression, virologic failure, drug resistance, and ART discontinuation or interruption, were outside the prespecified scope of this study.

## CONCLUSIONS

In a national cohort of PWH in Italy, transgender participants had a substantially higher risk of LTFU than cisgender participants, and this association remained after multivariable adjustment and in sensitivity analyses. These findings highlight persistent disparities in retention in HIV care among transgender people despite universal access to treatment. Analyses of major clinical outcomes were inconclusive because of the limited number of events. Systematic collection of gender identity data and the development of integrated, gender-affirming models of HIV care are essential to improve retention, continuity, and long-term health outcomes for transgender women living with HIV in Europe.

## Supplementary Material

ofag640_Supplementary_Data

## Supplementary Data

Supplementary materials are available at *Open Forum Infectious Diseases* online. Consisting of data provided by the authors to benefit the reader, the posted materials are not copyedited and are the sole responsibility of the authors, so questions or comments should be addressed to the corresponding author.
