# Supplementary material for: Higher Risk of Loss to Follow-up Among Transgender People With HIV in Italy: Findings From the ICONA Cohort
Source: Open Forum Infect Dis. 2026 Oct 8;13(10):ofag640. doi: 10.1093/ofid/ofag640 (PMC13660695; doi:10.1093/ofid/ofag640)
Supplement: Supplementary file 1 [file ofag640_supplementary_data.zip › Supplementary Figure S1.docx]

Supplementary Figure S1. Distribution of baseline CD4 cell counts according to gender identity. Histograms show the distribution of baseline CD4 cell counts (cells/mm³) among cisgender and transgender people with HIV (PWH). The upper panels include the overall study population, whereas the lower panels restrict the analysis to participants who initiated ART from 2016. Bars represent the percentage of participants within each CD4 count interval.

ALT text. Four-panel histogram showing the distribution of baseline CD4 cell counts in cisgender and transgender people with HIV. The upper row shows the overall population and the lower row participants initiated ART from 2016; cisgender participants are shown in the left panels and transgender participants in the right panels. CD4 counts are displayed on the x-axis and the percentage of participants on the y-axis. In all groups, most observations are concentrated below approximately 1,000 cells/mm³, with right-skewed distributions and a small number of higher CD4 values. The figure displays the complete observed distribution rather than only summary measures such as the median and interquartile range.
