# Supplementary material for: Higher Risk of Loss to Follow-up Among Transgender People With HIV in Italy: Findings From the ICONA Cohort
Source: Open Forum Infect Dis. 2026 Oct 8;13(10):ofag640. doi: 10.1093/ofid/ofag640 (PMC13660695; doi:10.1093/ofid/ofag640)
Supplement: Supplementary file 1 [file ofag640_supplementary_data.zip › Table S1 Characteristics 2016 R1.docx]

Table S1: Demographic and clinical characteristics at ART initiation according to gender identity, restricted to people who initiated ART from 2016 onward

| Variables | Total  n=5,820 | Transgender  n=173 | Cisgender n=5,647 | p-value |
| --- | --- | --- | --- | --- |
| Age (years), median (IQR) | 40 (31-50) | 32 (28-41) | 40 (31-50) | <0.0001 |
| Male sex assigned at birth, n (%) | 4811 (82.7) | 172 (99.4) | 4639 (82.2) | <0.0001 |
| Italian origin, n (%) | 4080 (70.1) | 12 (6.9) | 4068 (72.0) | <0.0001 |
| PWID, n (%) | 289 (5.0) | 4 (2.3) | 285 (5.1) | 0.102 |
| Smoking habit, n (%) |  |  |  | 0.239 |
| Yes | 2862 (49.2) | 96 (55.5) | 2766 (49.0) |  |
| No | 2209 (38.0) | 58 (33.5) | 2151 (38.1) |  |
| Unknown | 749 (12.9) | 19 (11.0) | 730 (12.9) |  |
| Comorbidities, n (%) |  |  |  |  |
| Previous AIDS diagnosis | 687 (11.8) | 12 (6.9) | 675 (12.0) | 0.044 |
| Previous MACE event | 39 (0.7) | 0 (0.0) | 39 (0.7) | 0.630 |
| Previous cancer diagnosis | 77 (1.3) | 1 (0.6) | 76 (1.4) | 0.729 |
| Previous kidney/liver event | 7 (0.1) | 0 (0.0) | 7 (0.1) | 1.000 |
| Previous diabetes | 123 (2.1) | 2 (1.2) | 121 (2.1) | 0.588 |
| Previous hypertension | 361 (6.2) | 5 (2.9) | 356 (6.3) | 0.066 |
| HCVAb positivity |  |  |  | 0.192 |
| Yes | 235 (4.0) | 3 (1.7) | 232 (4.1) |  |
| No | 4067 (69.9) | 129 (74.6) | 3938 (69.7) |  |
| Unknown | 1518 (26.1) | 41 (23.7) | 1477 (26.2) |  |
| HbsAg positivity |  |  |  | 0.274 |
| Yes | 168 (2.9) | 8 (4.6) | 160 (2.8) |  |
| No | 4198 (72.1) | 118 (68.2) | 4080 (72.3) |  |
| Unknown | 1454 (25.0) | 47 (27.2) | 1407 (24.9) |  |
| CD4, cells/mm3 median (IQR) | n=5598  319 (124-517) | n=171  402 (235-580) | n=5427  316 (121-514) | <0.0001 |
| CD4, cells/mm3 n (%) |  |  |  | <0.0001 |
| <200 | 1925/5598 (34.4) | 36/171 (21.1) | 1889/5427 (34.8) |  |
| 200-349 | 1118/5598 (20.0) | 28/171 (16.4) | 1090/5427 (20.1) |  |
| 350-499 | 1067/5598 (19.1) | 46/171 (26.9) | 1021/5427 (18.8) |  |
| ≥500 | 1488/5598 (26.6) | 61/171 (35.7) | 1427/5427 (26.3) |  |

The reported p-values are from the Wilcoxon Rank Sum test for continuous variables and Chi-Square test or Fisher’s Exact test for categorical variables. Missing values were treated as an “Unknown” category for categorical variables and reported as counts for continuous variables.
List of abbreviations: n, number; MACE; major cardiovascular events; IQR, inter quartile range; PWID, people who inject drugs.
