# Supplementary material for: Higher Risk of Loss to Follow-up Among Transgender People With HIV in Italy: Findings From the ICONA Cohort
Source: Open Forum Infect Dis. 2026 Oct 8;13(10):ofag640. doi: 10.1093/ofid/ofag640 (PMC13660695; doi:10.1093/ofid/ofag640)
Supplement: Supplementary file 1 [file ofag640_supplementary_data.zip › Table S2 con WvsW.docx]

Table S2: Demographic and clinical characteristics at enrolment according to gender identity, restricted to women.

| Variables | Transgender, woman  n=255 | Cisgender, woman n=1,748 | p-value |
| --- | --- | --- | --- |
| Age (years), median (IQR) | 32 (28-41) | 39 (31-49) | <0.001 |
| Italian origin, n (%) | 21 (8.2) | 895 (51.2) | <0.001 |
| PWID, n (%) | 6 (2.4) | 106 (6.1) | 0.016 |
| Smoking habit, n (%) |  |  | <0.001 |
| Yes | 136 (53.3) | 614 (35.1) |  |
| No | 96 (37.7) | 930 (53.2) |  |
| Unknown | 23 (9.0) | 204 (11.7) |  |
| Comorbidities, n (%) |  |  |  |
| Previous AIDS diagnosis | 24 (9.4) | 203 (11.6) | 0.300 |
| Previous MACE event | 0 (0.0) | 5 (0.3) | 0.393 |
| Previous cancer diagnosis | 1 (0.4) | 40 (2.3) | 0.046 |
| Previous kidney/liver event | - | - | - |
| Previous diabetes | 3 (1.2) | 19 (1.1) | 0.754 |
| Previous hypertension | 6 (2.4) | 100 (5.7) | 0.025 |
| HCVAb positivity |  |  | 0.078 |
| Yes | 9 (3.5) | 104 (6.0) |  |
| No | 239 (93.7) | 1558 (89.1) |  |
| Unknown | 7 (2.8) | 86 (4.9) |  |
| HbsAg positivity |  |  | 0.017 |
| Yes | 15 (5.9) | 46 (2.6) |  |
| No | 232 (91.0) | 1635 (93.5) |  |
| Unknown | 8 (3.1) | 67 (3.8) |  |
| Year of enrolment, n (%) |  |  | 0.003 |
| 2010-2015 | 78 (30.6) | 706 (40.4) |  |
| 2016-2023 | 177 (69.4) | 1042 (59.6) |  |
| CD4, cells/mm3 median (IQR) | n=245  412 (237-581) | n=1627  308 (125-519) | <0.001 |
| CD4, cells/mm3 n (%) |  |  | <0.001 |
| <200 | 53/245 (21.6) | 568/1627 (34.9) |  |
| 200-349 | 38/245 (15.5) | 342/1627 (21.0) |  |
| 350-499 | 64/245 (26.1) | 285/1627 (17.5) |  |
| ≥500 | 90/245 (36.7) | 432/1627 (26.6) |  |

The reported p-values are from the Wilcoxon Rank Sum test for continuous variables and Chi-Square test or Fisher’s Exact test for categorical variables. Missing values were treated as an “Unknown” category for categorical variables and reported as counts for continuous variables.
List of abbreviations: n, number; MACE; major cardiovascular events; IQR, inter quartile range; PWID, people who inject drugs.
