# Supplementary material for: Higher Risk of Loss to Follow-up Among Transgender People With HIV in Italy: Findings From the ICONA Cohort
Source: Open Forum Infect Dis. 2026 Oct 8;13(10):ofag640. doi: 10.1093/ofid/ofag640 (PMC13660695; doi:10.1093/ofid/ofag640)
Supplement: Supplementary file 1 [file ofag640_supplementary_data.zip › Table S3 Characteristics 2016vsOther.docx]

Table S3: Demographic and clinical characteristics at ART initiation among participants from centres included versus excluded from the present analysis, restricted to people who initiated ART from 2016 onward

| Variables | Participants from included centers  n=5,820 | Participants from excluded centers n=2,003 |
| --- | --- | --- |
| Age (years), median (IQR) | 40 (31-50) | 41 (32-50) |
| Male sex assigned at birth, n (%) | 4811 (82.7) | 1579 (78.8) |
| Italian origin, n (%) | 4080 (70.1) | 1456 (72.7) |
| PWID, n (%) | 289 (5.0) | 122 (6.1) |
| Smoking habit, n (%) |  |  |
| Yes | 2862 (49.2) | 962 (48.0) |
| No | 2209 (38.0) | 664 (33.2) |
| Unknown | 749 (12.9) | 377 (18.8) |
| Comorbidities, n (%) |  |  |
| Previous AIDS diagnosis | 687 (11.8) | 250 (12.5) |
| Previous MACE event | 39 (0.7) | 19 (1.0) |
| Previous cancer diagnosis | 77 (1.3) | 31(1.6) |
| Previous kidney/liver event | 7 (0.1) | 3 (0.2) |
| Previous diabetes | 123 (2.1) | 46 (2.3) |
| Previous hypertension | 361 (6.2) | 124 (6.2) |
| HCVAb positivity |  |  |
| Yes | 235 (4.0) | 73 (3.6) |
| No | 4067 (69.9) | 1256 (62.7) |
| Unknown | 1518 (26.1) | 674 (33.7) |
| HbsAg positivity |  |  |
| Yes | 168 (2.9) | 66 (3.3) |
| No | 4198 (72.1) | 1320 (65.9) |
| Unknown | 1454 (25.0) | 617 (30.8) |
| CD4, cells/mm3 median (IQR) | n=5598  319 (124-517) | n=1933  305 (101-514) |
| CD4, cells/mm3 n (%) |  |  |
| <200 | 1925/5598 (34.4) | 697/1933 (36.1) |
| 200-349 | 1118/5598 (20.0) | 371/1933 (19.2) |
| 350-499 | 1067/5598 (19.1) | 354/1933 (18.3) |
| ≥500 | 1488/5598 (26.6) | 511/1933 (26.4) |

P-values are not reported because this was an exploratory comparison and, given the large sample sizes, even small differences could reach statistical significance despite limited clinical relevance. Missing values were treated as an “Unknown” category for categorical variables and reported as counts for continuous variables.
List of abbreviations: n, number; MACE; major cardiovascular events; IQR, inter quartile range; PWID, people who inject drugs.
