# Supplementary material for: Higher Risk of Loss to Follow-up Among Transgender People With HIV in Italy: Findings From the ICONA Cohort
Source: Open Forum Infect Dis. 2026 Oct 8;13(10):ofag640. doi: 10.1093/ofid/ofag640 (PMC13660695; doi:10.1093/ofid/ofag640)
Supplement: Supplementary file 1 [file ofag640_supplementary_data.zip › Table S4 Definite LTFU.docx]

Supplementary Table 4. Proportion of temporary and definitive LTFU.

|  | Loss to  follow-up, n | Definitive loss, n (%) | | | Non-definitive loss, n (%) | | | p-value |
| --- | --- | --- | --- | --- | --- | --- | --- | --- |
| Population | **Total** | **Total*** | **Transgender**** | **Cisgender**** | **Total*** | **Transgender**** | **Cisgender**** |  |
| Primary set (N=10,178) | 4026 | 2761 (68.6) | 96 (70.6) | 2665 (68.5) | 1265 (31.4) | 40 (29.4) | 1225 (31.5) | 0.697 |
| ART from 2016 onward (n=5,820) | 1764 | 1303 (73.9) | 51 (69.9) | 1252 (74) | 461 (26.1) | 22 (30.1) | 439 (26) | 0.426 |

The reported p-values are from the Chi-Square test; bold values are significant at the selected level of significance (α = 0.05).
*Percentages based on the subgroup of patients with loss to follow-up
**Percentages based on the relative subgroup of patients with definitive/non-definitive loss
