# Supplementary material for: Higher Risk of Loss to Follow-up Among Transgender People With HIV in Italy: Findings From the ICONA Cohort
Source: Open Forum Infect Dis. 2026 Oct 8;13(10):ofag640. doi: 10.1093/ofid/ofag640 (PMC13660695; doi:10.1093/ofid/ofag640)
Supplement: Supplementary file 1 [file ofag640_supplementary_data.zip › Table S5 Additional Multivariable models.docx]

|  | Multivariable | |
| --- | --- | --- |
| Overall^*^ | **sHR^^^ (95% CI)** | **p-value** |
| Transgender woman (vs. Cisgender woman) | 1.27 (1.06-1.53) | 0.012 |
| Restricted to 2016 onward^°^ | **sHR^§^ (95% CI)** | **p-value** |
| Transgender woman (vs. Cisgender woman) | 1.24 (0.96-1.59) | 0.098 |

Table S5: Additional multivariable models to assess the effect of gender identity on risk of loss to follow-up.

The reported p-values are from the Fine-Gray regression analysis with death as competing event.

*878 events

°395 events

^^^Adjusted for years of enrolement (2010-2015 vs 2016-2023), age, previous AIDS diagnosis, country of birth and for people who inject drugs.

^§^Adjusted for age, previous AIDS diagnosis, country of birth and for people who inject drugs.

List of abbreviations: sHR: sub-distribution Hazard Ratio; CI: Confidence Interval.
