# Supplementary material for: Higher Risk of Loss to Follow-up Among Transgender People With HIV in Italy: Findings From the ICONA Cohort
Source: Open Forum Infect Dis. 2026 Oct 8;13(10):ofag640. doi: 10.1093/ofid/ofag640 (PMC13660695; doi:10.1093/ofid/ofag640)
Supplement: Supplementary file 1 [file ofag640_supplementary_data.zip › Table S6 Breakdown of clinical outcomes-Overall_R1.docx]

Supplementary Table 6. Breakdown of clinical outcome in the overall study population.

|  | AIDS | Cancer | MACE | Kidney-Liver disease  (non-AIDS) | Death | Total |
| --- | --- | --- | --- | --- | --- | --- |
| Cisgender PWH | 322 (34.1%) | 210 (22.2%) | 56 (5.9%) | 19  (2%) | 337 (35.7%) | **944** |
| Transgender PWH | 9 (52.9%) | 1 (5.9%) | 2 (11.8%) | 0 (0%) | 5 (29.4%) | **17** |
| Total | **331** | **211** | **58** | **19** | **342** | **961** |
| Cisgender woman with HIV | 64  (40.5%) | 39  (24.7%) | 3  (1.9%) | 3  (1.9%) | 49  (31.0%) | **158** |
