# Supplementary material for: Higher Risk of Loss to Follow-up Among Transgender People With HIV in Italy: Findings From the ICONA Cohort
Source: Open Forum Infect Dis. 2026 Oct 8;13(10):ofag640. doi: 10.1093/ofid/ofag640 (PMC13660695; doi:10.1093/ofid/ofag640)
Supplement: Supplementary file 1 [file ofag640_supplementary_data.zip › Table S7 Breakdown of clinical outcomes-Post 2016_R1.docx]

Supplementary Table 7. Breakdown of clinical outcomes among PWH who initiated ART from 2016.

|  | AIDS | Cancer | MACE | Kidney-Liver disease  (non-AIDS) | Death | Total |
| --- | --- | --- | --- | --- | --- | --- |
| Cisgender PWH | 200 (47.4%) | 68 (16.1%) | 12 (2.8%) | 5 (1.2%) | 137 (32.5%) | **422** |
| Transgender PWH | 9 (81.8%) | 1 (9.1%) | 0 (0%) | 0 (0%) | 1 (9.1%) | **11** |
| Total | **209** | **69** | **12** | **5** | **138** | **433** |
| Cisgender WWH | 39  (50.7%) | 14  (18.2%) | 0  (0.0%) | 1  (1.3%) | 23  (29.9%) | **77** |
