# Supplementary material for: Higher Risk of Loss to Follow-up Among Transgender People With HIV in Italy: Findings From the ICONA Cohort
Source: Open Forum Infect Dis. 2026 Oct 8;13(10):ofag640. doi: 10.1093/ofid/ofag640 (PMC13660695; doi:10.1093/ofid/ofag640)
Supplement: Supplementary file 1 [file ofag640_supplementary_data.zip › Table S8 additional models outcome.docx]

Table S8: Additional multivariable models to assess the effect of gender identity on the risk of a composite clinical outcome composed by mortality, major adverse cardiovascular events (MACE), cancer incidence, AIDS progression, and other severe non-AIDS events.

|  | Multivarianble | |
| --- | --- | --- |
| Overall^*^ | **aHR^^^**  **(95% CI)** | **p-value** |
| Transgender woman (vs. Cisgender woman) | 1.26 (0.74-2.16) | 0.396 |
| Restricted to 2016 onward^°^ | **aHR^§^**  **(95% CI)** | **p-value** |
| Transgender woman (vs. Cisgender woman) | 1.49 (0.75-2.97) | 0.259 |

The reported p-values are from the Cox regression analysis.

*175 events

°88 events

^^^Adjusted for years of enrolement (2010-2015 vs 2016-2023), age, previous AIDS diagnosis, previous MACE, previous cancer, previous kidney/liver event, previous diabetes and hypertension, CD4 count at enrolement, country of birth, HCV and HBsAg positivity, smoking habits and for people who inject drugs.

^§^Adjusted for age, previous AIDS diagnosis, previous MACE, previous cancer, previous kidney/liver event, previous diabetes and hypertension, CD4 count at enrolement, country of birth, HCV and HBsAg positivity, smoking habits and for people who inject drugs.

List of abbreviations: aHR: Adjusted Hazard Ratio; HR: Hazard Ratio; CI: Confidence Interval.
